# Undetected cases after implementation of first‐trimester anomaly scan in low‐risk population: insights from the IMITAS study

**DOI:** 10.1002/uog.70131

**Published:** 2025-11-14

**Authors:** K. Bronsgeest, E. E. R. Lust, S. C. E. Zaaijer, L. Henneman, N. Crombag, C. M. Bilardo, R.‐J. H. Galjaard, E. Sikkel, A. K. K. Teunissen, M. N. Bekker, M. C. Haak, Collaborators, A. B. C. Coumans, A. Elvan‐Taşpınar, S. Galjaard, A. T. J. I. Go, E. van Leeuwen, G. T. R. Manten, E. Pajkrt

**Affiliations:** ^1^ Department of Obstetrics and Gynecology Leiden University Medical Center Leiden The Netherlands; ^2^ Department of Obstetrics and Gynecology University Medical Center Utrecht Utrecht The Netherlands; ^3^ Department of Human Genetics Amsterdam UMC, location Vrije Universiteit Amsterdam Amsterdam The Netherlands; ^4^ Amsterdam Reproduction and Development Research Institute, Amsterdam UMC, location Vrije Universiteit Amsterdam Amsterdam The Netherlands; ^5^ Department of Obstetrics and Gynecology Amsterdam UMC Amsterdam The Netherlands; ^6^ Department of Clinical Genetics Erasmus University Medical Center Rotterdam The Netherlands; ^7^ Department of Obstetrics and Gynecology Radboud University Medical Center Nijmegen The Netherlands

**Keywords:** early diagnosis, fetal anomaly, fetal screening, prenatal detection, prenatal ultrasonography

## Abstract

**Objective:**

To assess the effectiveness of the first‐trimester anomaly scan (FTAS) performed as part of a centrally steered national screening program in The Netherlands by investigating false‐negative cases with a fetal structural anomaly that was not detected at the FTAS.

**Methods:**

This was a secondary analysis of the IMplementation of fIrst Trimester Anomaly Scan (IMITAS) study, a national prospective cohort study conducted in a low‐risk population in The Netherlands between November 2021 and November 2022. The FTAS was performed according to a predefined scanning protocol. We collected all cases with a fetal structural anomaly that was not detected at the FTAS and was diagnosed at a tertiary care center before 24 weeks' gestation following a referral based on the second‐trimester anomaly scan. These anomalies were classified as: (1) ‘always detectable’ in the first trimester (e.g. holoprosencephaly, anencephaly), referred to as first‐trimester major anomalies (FTMAs); (2) ‘often detectable’ in the first trimester (e.g. spina bifida, major heart defect); or (3) ‘undetectable’ in the first trimester (e.g. agenesis of the corpus callosum). To investigate the reasons for undetected FTMA, sonographic images from these cases and from a sample of normal controls were retrieved and scored for quality by two fetal medicine experts, who were blinded to the fetal outcome.

**Results:**

Of the 127 979 cases with a normal FTAS result, 1164 (0.9%) had a fetal structural anomaly diagnosed before 24 weeks' gestation. Seven cases could not be categorized because intrauterine fetal death occurred before arrival at the tertiary care center. Among the 1157 remaining cases with an undetected anomaly, 23 (2.0%) had a FTMA, comprising mainly limb reduction defects, hydrops and megacystis, with seven cases of multiple congenital anomalies. A further 126 (10.9%) cases had an anomaly that is considered often detectable in the first trimester, which primarily comprised cardiac defects and central nervous system anomalies. Lastly, 1008 (87.1%) cases had an anomaly that is considered undetectable in the first trimester, represented mainly by urogenital and cardiac anomalies. Quality assessment of images obtained at the FTAS in 23 cases with undetected FTMA and 19 controls showed that the quality score for fetal anatomy was significantly lower in cases *vs* controls (*P* = 0.003), with mandatory planes missing more often (*P* = 0.039).

**Conclusions:**

Nearly 1% of pregnancies with a normal FTAS result were diagnosed with a structural anomaly in the second trimester, including 23 major anomalies amenable to detection in the first trimester (accounting for 0.02% of scans with a normal result). Missing mandatory planes and acquisition of suboptimal planes were the main reasons for the overlooked FTMA diagnoses. Improvements are expected with increasing sonographer experience, additional training and possible expansion of the protocol. © 2025 The Author(s). *Ultrasound in Obstetrics & Gynecology* published by John Wiley & Sons Ltd on behalf of International Society of Ultrasound in Obstetrics and Gynecology.

## INTRODUCTION

In developed countries, a second‐trimester anomaly scan is offered between 18 and 24 weeks' gestation for the detection of fetal structural anomalies^1^. However, a substantial proportion of major congenital anomalies can be detected earlier, and an early scan has been suggested as a means to empower prospective parents with more reproductive autonomy^2^, ^3^. Detection rates in the first trimester are reported to be near 100% for major congenital anomalies, such as anencephaly, encephalocele, holoprosencephaly, megacystis and body‐stalk anomaly^4^, ^5^, ^6^, ^7^. However, the efficacy of first‐trimester screening for structural anomalies may be context‐dependent. A systematic review of first‐trimester ultrasound screening showed significant variation in sensitivity for all types of fetal anomaly, ranging from 32% in low‐risk or unselected populations to 61% in high‐risk groups^2^. It is essential to acknowledge, however, that the majority of studies on this subject have been conducted in a secondary or tertiary care setting, prompting questions about the performance of first‐trimester screening in primary care^2^. In The Netherlands, a first‐trimester anomaly scan (FTAS) was offered as part of a national implementation study, the IMplementation of fIrst Trimester Anomaly Scan (IMITAS) study, with the aim of detecting major structural anomalies earlier in pregnancy to inform parents' reproductive choices and enhance decision‐making^8^. A considerable number of important anomalies were not detected in this program^8^. Discerning the type and severity of the anomalies that remained undetected, and finding possible explanations for their lack of detection, are essential to steer the program and improve quality in the future. Thus, the aim of this study was to describe the type of anomalies that were not detected at the FTAS, offered as part of a national screening program in The Netherlands. Additionally, we sought to identify possible reasons why these cases remained undetected by studying the images and scan reports.

## METHODS

This was a secondary analysis of the IMITAS study, a prospective, observational national cohort study conducted in The Netherlands^8^. The FTAS standardized screening protocol is summarized in Table 1.

**Table 1:** Standardized screening protocol for first‐trimester anomaly scan in The Netherlands^8^, ^16^

Criterion
General characteristics
Number of fetuses
Viability
Volume of amniotic fluid
Placental localization
Fetal growth parameters
Crown–rump length
Head circumference
Abdominal circumference
Femur length
Fetal anatomy: mandatory planes
Skull: intactness and shape
Brain: visualization of midline and choroid plexus
Neck: NT measurement
Spine
Visualization of spine in sagittal plane
Continuity of skin
Face: profile
Thorax
Shape of thorax
Aspect of lungs
Heart
Position of heart
Four‐chamber view
Abdomen
Abdominal wall and umbilical cord insertion
Stomach and bladder filling
LBD measurement
Extremities
Presence of both upper and lower extremities
Presence of both hands
Presence of both feet

LBD, longitudinal bladder diameter; NT, nuchal translucency.

### Patient inclusion and follow‐up

Cases with a structural anomaly that was not detected at the FTAS between 1 November 2021 and 1 November 2022 were extracted from the electronic patient databases of all eight tertiary care centers for fetal medicine across The Netherlands. Follow‐up was conducted by utilizing the prospective databases of each center to identify cases referred for a detailed diagnostic scan between 18 and < 24 weeks' gestation during the study period. The ultrasound reports were retrieved from the electronic patient files and checked manually. Cases were included when the referral was based on an anomaly suspected at the second‐trimester anomaly scan. To categorize the defect, we used the scan report from the last detailed diagnostic scan conducted < 24 weeks' gestation. Furthermore, we collected the results of genetic testing and pregnancy outcome. Cases with isolated abnormal fetal growth without a genetic aberration, those with a second‐trimester sonomarker (e.g. single umbilical artery, increased bowel echogenicity) and those with an amniotic fluid or placental anomaly were not included in this analysis. We excluded women who opted not to undergo a FTAS.

### Outcome

The scan report of each case was reviewed by a fetal medicine specialist (M.C.H.) who is an expert in fetal ultrasound, and then categorized by organ system. All cases were allocated to one of three groups. The first group comprised first‐trimester major anomalies (FTMAs), defined as anomalies that are expected to be always detectable in the first trimester, as described in our previous study^8^, namely anencephaly, encephalocele, holoprosencephaly, abdominal wall defect, body‐stalk anomaly, megacystis, bladder exstrophy, limb reduction defect, hydrops fetalis and twin reversed arterial perfusion sequence. The second group comprised anomalies that are often detectable in the first trimester, such as complex (mostly univentricular) heart defects, other cardiac anomalies resulting in an abnormal four‐chamber view (4CV) (including unbalanced atrioventricular septal defect (AVSD)), balanced AVSD, hydrocephalus, spina bifida, diaphragmatic hernia, pulmonary anomaly, retrognathia, micrognathia and certain forms of skeletal dysplasia. The third group comprised anomalies that are not expected to be detected in the first trimester (‘undetectable’), either because the Dutch national FTAS protocol does not include assessment of that anatomical plane (e.g. fetal lips) or the anomaly is not yet visible in the first trimester (e.g. agenesis of the corpus callosum)^3^.

Multiple congenital anomalies (MCA) were defined as two or more anomalies observed in more than one organ system. Cases with more than one structural anomaly in the same organ system were classified according to the most severe anomaly. Two cases each with two minor structural anomalies (persistent right umbilical vein (PRUV) and aberrant right subclavian artery in one case, PRUV and mesocardia in the other) were not considered as MCA, but categorized as a single structural anomaly (vascular anomaly).

### Image assessment

Images obtained at the FTAS were retrieved for cases with an undetected FTMA, as well as for a similar number of controls from the same period from four midwifery practices across The Netherlands. Controls had both a normal FTAS and a normal second‐trimester anomaly scan. The cases were anonymized and put in a random order. Two fetal tertiary care experts (A.K.K.T., M.C.H.) assessed the images but were blinded to the outcome of the cases (i.e. control *vs* undetected FTMA, and type of anomaly). The two experts jointly reviewed and scored the images, reaching consensus on each case, to identify potential systematic factors, such as image quality and technical performance. Availability of mandatory planes, correctness of the planes and image quality were scored using a structured evaluation form which was developed for quality monitoring of the FTAS screening program in The Netherlands (Appendix S1). The maximum score was 55 points: 41 points were available for fetal anatomy and 14 for fetal biometry. Missing planes were counted and 0 points were allocated for the anatomy in that plane. Image resolution was scored as good or poor. Additionally, the experts reported in a free‐text field if they suspected or identified any (potential) fetal anomalies.

### Statistical analysis

Normally distributed continuous variables are presented as mean ± SD, while non‐normally distributed continuous variables are presented as median (interquartile range (IQR)). Categorical variables are presented as *n* (%). Detection rates were calculated for FTMA and several anomalies of the often‐detectable group. Multiple logistic regression analysis was performed to evaluate the association between maternal/pregnancy characteristics and the detection of FTMA at the FTAS. Statistical analysis was performed using SPSS version 25 (IBM Corp., Armonk, NY, USA). Results were considered significant at *P* < 0.05.

## RESULTS

Overall, 129 704 pregnancies underwent a FTAS during the study period^8^. Among these, 332 structural anomalies, 117 genetic anomalies and 82 other anomalies (abnormal fetal biometry, second‐trimester sonomarker, placental/umbilical cord anomaly, an‐/oligohydramnios) were detected, corresponding to an overall sensitivity of 31.6% for all types of anomaly and 84.6% specifically for FTMA^8^.

Of 127 979 cases classified as normal at the FTAS, a fetal structural anomaly was later diagnosed before 24 weeks' gestation in 1164 (0.9%) cases. Table 2 summarizes the baseline characteristics of these cases and their pregnancy outcome. Seven cases could not be categorized into one of the three groups (FTMA, often‐detectable anomaly or undetectable anomaly in the first trimester) because they underwent intrauterine fetal death (IUFD) before arrival at the fetal medicine center, therefore the anomaly could not be defined conclusively. We therefore categorized 1157 undetected anomalies.

**Table 2:** Baseline characteristics, prenatal diagnosis and outcome of 1164 pregnancies with fetal structural anomaly that was not detected at first‐trimester anomaly scan

Parameter	Value (*n* = 1164)
Maternal age (years)	31.6 ± 4.6
Prepregnancy BMI* (kg/m^2^)	24 (21–27)
Nulliparous	566 (48.6)
Singleton	1143 (98.2)
GA at first‐trimester anomaly scan (weeks)	13 + 4 ± 0 + 4
GA at second‐trimester anomaly scan (weeks)	19 + 4 ± 0 + 4
Prenatal diagnosis†	
First‐trimester major anomaly	23/1157 (2.0)
Often‐detectable anomaly in first trimester	126/1157 (10.9)
Undetectable anomaly in first trimester	1008/1157 (87.1)
Pregnancy outcome	
Live birth	952 (81.8)
TOP	180 (15.5)
IUFD	23 (2.0)
Other‡	9 (0.8)
NICU admission§	217/952 (22.9)
Neonatal death§	16/952 (1.7)

Data are given as mean ± SD, median (interquartile range), *n* (%) or *n*/*N* (%).

* Data missing for 18 patients.

† Data missing for seven cases that underwent intrauterine fetal demise (IUFD) before arrival at fetal medicine center.

‡ Preterm delivery < 24 weeks, *n* = 1; lost to follow‐up, *n* = 8.

§ Denominator is number of live births. BMI, body mass index; GA, gestational age; NICU, neonatal intensive care unit; TOP, termination of pregnancy.

### First‐trimester major anomalies

In total, 23 women with a normal FTAS result were diagnosed with a FTMA before 24 weeks' gestation, accounting for 0.02% of the 127 979 cases with a normal FTAS result and 2.0% of the 1157 cases with an undetected anomaly. Of those, 56.5% underwent IUFD or termination of pregnancy (TOP). A genetic aberration was diagnosed in two cases, namely trisomy 21 and tuberous sclerosis complex.

The 23 cases are classified by organ system in Table 3, along with the 126 FTMA cases that were detected successfully at the FTAS. In 7/23 undetected cases, MCA were present. Using multiple logistic regression analysis, detection of FTMA at the FTAS was not found to be associated significantly with maternal body mass index (BMI) (*P* = 0.631), gestational age at the FTAS (*P* = 0.369) or maternal age (*P* = 0.567).

**Table 3:** Detection of first‐trimester major anomalies at first‐trimester anomaly scan

	Undetected (*n*)			
Structural anomaly	Total	Part of MCA	Detected (*n*)	Detection rate (%)
Anencephaly	0	—	8	100
Encephalocele	2	1	6	75.0
Holoprosencephaly	2	2	11	84.6
Abdominal wall defect*	2	2	40	95.2
Body‐stalk anomaly	0	—	0	—
Megacystis	4	0	16	80.0
Bladder exstrophy	1	0	1	50.0
Limb reduction defect	6	1	17	73.9
Hydrops	5	1	24	82.8
TRAP sequence	1	NA	3	75.0

* Omphalocele, gastroschisis. MCA, multiple congenital anomalies; NA, not applicable; TRAP, twin reversed arterial perfusion.

The most frequently undetected FTMA was limb reduction defect (*n* = 6), all instances of which were upper extremity defects. Of the two cases of undetected abdominal wall defect, one had a small omphalocele combined with a unilateral hydroureter and the other had a ventricular septal defect, short ribs, pectus excavatum, shortened long bones, bowed femur and multiple cysts in the placenta next to the omphalocele. The maternal BMI of the latter case was 27 kg/m^2^. There were two cases with undetected holoprosencephaly; when the scan reports were studied, the holoprosencephaly was determined to be semilobar. One of the two cases with encephalocele had a small occipital defect at a gestational age of 21 + 2 weeks. The other case had a large encephalocele and MCA, including severe hydrops, anhydramnios, abnormal thorax/abdomen ratio, unilateral multicystic kidney dysplasia and possible abnormal extremities.

#### Image assessment

Table 4 summarizes the quality assessment of images derived from the FTAS in 23 fetuses with undetected FTMA and 19 controls. The overall quality score was significantly lower in cases with undetected FTMA (median, 45 (IQR, 37–51) points *vs* 50 (IQR, 49–52) points; *P* = 0.014). In such cases, points were lost for the assessment of fetal anatomy due to the use of an incorrect plane for demonstrating a particular structure. Specifically, across undetected FTMA cases and controls, the correct midsagittal plane for evaluating the spine was not achieved in more than half of pregnancies, and the correct 4CV was obtained in only 66.7%. Furthermore, mandatory planes were missing more frequently in the FTMA group (12/23 (52.2%) *vs* 4/19 (21.1%); *P* = 0.039). In only 6/23 undetected FTMA cases, the experts observed an abnormality: two had an abdominal wall defect, while the type of anomaly could not be classified in the remaining four cases due to the inadequacy of the planes used for the examination. In 3/4 cases of megacystis, the bladder appeared normal on the available images; in one case, the abdominal plane was missing, precluding assessment of bladder size. In all five hydrops cases, the required planes were present and the images were indicative of normal anatomy.

**Table 4:** Quality assessment of images derived from first‐trimester anomaly scan in fetuses with undetected first‐trimester major anomaly (FTMA) and control fetuses

Parameter	Undetected FTMA (*n* = 23)	Controls (*n* = 19)	*P*
GA at first‐trimester anomaly scan (weeks)	13 + 4 ± 0 + 4	13 + 4 ± 0 + 3	0.811
Number of images per patient	34 (32–47)	37 (34–47)	0.239
Good image resolution	18 (78.3)	15 (78.9)	0.957
Total score*	45 (37–51)	50 (49–52)	0.014
Score for fetal anatomy	31 (25–37)	37 (35–41)	0.003
Score for fetal biometry	14 (12–14)	14 (12–14)	0.989
≥ 1 mandatory anatomical plane missing	12 (52.2)	4 (21.1)	0.039
Suspected fetal anomaly	6 (26.1)	2 (10.5)†	0.201

Data are given as mean ± SD, median (interquartile range) or *n* (%).

* Maximum score was 55 points: 41 points were available for fetal anatomy and 14 for fetal biometry.

† Case 1: multiple suboptimal planes, image resolution sufficient, choroid plexus only visible in posterior horn, possible widening of spine in sacral coronal plane; Case 2: strawberry skull. GA, gestational age.

### Often‐detectable anomalies in the first trimester

In total, 126/1157 (10.9%) anomalies undetected at the FTAS were classed as often detectable in the first trimester. These were mainly cardiac defects and anomalies of the central nervous system (Table 5). In more than half of the cases, the parents opted for TOP, indicating that the abnormalities in this group were severe. In 26/126 cases, invasive testing revealed a genetic aberration (Table S1).

**Table 5:** Classification of 126 fetuses with structural anomaly that is often detectable in the first trimester that was not detected at first‐trimester anomaly scan, according to pregnancy outcome

Structural anomaly	Total (*n* = 126)	Live birth (*n* = 46)	TOP (*n* = 76)	IUFD (*n* = 4)
Central nervous system	35	7	28	0
Spina bifida	30	7	23	0
Hydrocephalus	5	0	5	0
Face*	4	2	2	0
Thorax/lungs	14	9	5	0
Diaphragmatic hernia	10	6	4	0
Pulmonary anomaly†	4	3	1	0
Heart	41	17	21	3
Anomaly resulting in asymmetric 4CV‡	22	8	12	2
Complex defect resulting in abnormal 4CV§	8	1	6	1
Septal defect¶	9	6	3	0
Minor CHD**	2	2	0	0
Skeletal††	5	1	4	0
Ascites	3	1	2	0
Genetic syndrome after referral for abnormal biometry‡‡	4	1	3	0
Multiple congenital anomalies	20	8	11	1

Counts indicate number of fetuses with only that anomaly.

* Retrognathia, micrognathia, midface hypoplasia.

† Congenital high airway obstruction syndrome, hydrothorax.

‡ Hypoplastic left heart syndrome, hypoplastic right heart syndrome, Ebstein's anomaly, tricuspid hypoplasia, unbalanced atrioventricular septal defect (AVSD).

§ Isomerism with AVSD, abnormal position of fetal heart with intracardiac anomaly or complex outflow tract anomaly with asymmetric four‐chamber view (4CV).

¶ Balanced AVSD.

** Cardiac dextroposition.

†† Skeletal dysplasia.

‡‡ Trisomy 21 (*n* = 1), Wolf–Hirschhorn syndrome (*n* = 1), 13q34 deletion (*n* = 1) and *IGF1R* deletion (*n* = 1). CHD, congenital heart disease; IUFD, intrauterine fetal death; TOP, termination of pregnancy.

Spina bifida remained undetected in 31/58 cases (of which one had MCA), resulting in a detection rate of 46.6%. On review of the scan reports, only five of the undetected cases were small sacral defects; the other 26 were large defects located higher up the spine (lumbar and thoracic). Skeletal dysplasia was undetected in 7/16 cases (detection rate, 56.3%), of which two were MCA and at least four were lethal; at 20 weeks' gestation, these four fetuses demonstrated severely shortened and deformed limbs, and one case had an increased nuchal fold of 9 mm. The FTAS did not detect 10/22 complex cardiac defects (detection rate, 54.5%), of which two were MCA. Furthermore, 39/97 anomalies resulting in an asymmetric 4CV were undetected, of which 17 were MCA, and all cases of balanced AVSD were missed, resulting in a combined detection rate of 59.8%. The 10 undetected diaphragmatic hernias were all left‐sided. Of the seven cases of undetected hydrocephalus (including two cases with MCA), six were severe.

### Undetectable anomalies in the first trimester

The vast majority (1008/1157 (87.1%)) of the fetal structural anomalies diagnosed at the second‐trimester anomaly scan were considered to be undetectable in the first trimester (Table S2). These were predominantly urogenital anomalies, including ureteral duplication, pelvic kidney, horseshoe kidney, unilocular cyst and hydronephrosis. The cardiac defects were mainly outflow tract anomalies and septal defects; these could not be detected at the FTAS because of later development of the anomaly or because the ultrasound planes in which they are visible were not part of our protocol. In 52/1008 cases (5.2%), a genetic aberration was found (Table S3). Among all cases with an undetectable anomaly in the first trimester, the live birth rate was 88.9%.

## DISCUSSION

### Main findings

During the first year following nationwide implementation of the FTAS in The Netherlands, 1164/129 704 fetuses (0.9%) had a structural anomaly that was not detected at the FTAS. Of these, 23 (2.0%) had a FTMA, which should have been detected at the FTAS, and 126 (10.9%) had an anomaly that is often detectable in the first trimester. Cardiac defects and urogenital anomalies were the most frequently overlooked of all anomalies.

A significant proportion of women opted for TOP following the detection of the anomaly in the second trimester, underscoring the clinical relevance of timely diagnosis. Notably, this occurred in a setting characterized by uniformly trained sonographers adhering to a standardized protocol and subject to biannual quality monitoring. The second‐trimester anomaly scan in The Netherlands is well‐established and considered of high quality^9^, ^10^, ^11^. Understanding why FTMA and other (major) anomalies remained undetected is essential, particularly for expectation management of prospective parents, quality improvement, training of sonographers and/or protocol adjustment. Assessment of the FTAS images from FTMA cases showed that missing mandatory planes (incomplete scan) and acquisition of suboptimal planes by the sonographer were the main explanations for failed anomaly detection, suggesting a primary underlying difficulty in technical execution rather than interpretation. The absence of mandatory planes suggests poor protocol adherence, potentially due to difficulty in plane acquisition, lack of operator motivation or shortage of time allocated for the scan. Further investigation is warranted to understand the underlying reasons for this finding to support program improvement. Additionally, a remarkable number of large neural tube defects and heart defects that are clearly visible in the 4CV were not detected. Although these cases were not included in the image evaluation, it is plausible that similar factors played a role in their failed identification as it proved challenging to acquire the exact midsagittal plane of the spine or the correct transverse thoracic plane to visualize the 4CV in the evaluation of FTMA cases. Recognition of abnormal anatomy in the first trimester might be difficult, as two omphaloceles were visible in the stored planes on image assessment, and even cases of MCA were overlooked. The undetected encephaloceles suggest that sonographers may have focused on acquiring the mandatory planes rather than examining the entire fetus. These results demonstrate that, as observed with the second‐trimester anomaly scan^12^, the test characteristics of population‐based screening are not comparable to the performance achieved in tertiary diagnostic centers.

### Research implications

The detection rates for certain major anomalies, such as spina bifida and univentricular cardiac defects, were considerably lower compared with previous reports^2^, ^3^. This may be explained by the fact that most other studies have been executed in settings with a specific interest in first‐trimester ultrasound screening. This differs from a national primary care setting involving many sonographers, who, although skilled and uniformly trained, were often working independently in small practices^2^, ^3^, ^7^. Furthermore, some FTMAs might have been identified already during the dating scan, as indicated by the absence of body‐stalk anomalies and the low incidence of anencephaly, which may hamper comparison with previous reports. A detailed evaluation of the factors contributing to anomalies being undetected in the FTAS is essential to improve diagnosis in the future. It remains uncertain as to whether sonographer dexterity, years of experience, training or the FTAS protocol itself has the greatest impact. The discrepancy in spina bifida detection can also be attributed to our protocol, which did not include obtaining a midsagittal section of the brain for examining the posterior fossa^13^, ^14^. Similar reasoning applies to univentricular heart defects, as our protocol only mandates evaluation and acquisition of the 4CV and does not require referral in case of a suboptimal plane if the sonographer was convinced of normality^3^, ^15^. However, the effect of implementing additional (advanced) planes in a first‐trimester screening setting, such as outflow tracts or intracranial translucency for the detection of spina bifida, is unknown. Besides an expected higher detection rate, this might lead to more false positives^2^. Thus, changes in the scan protocol should be studied carefully to ensure that the desired effect is achieved.

### Clinical implications

Early screening and diagnosis are important to allow prospective parents more time to undergo further testing and make informed reproductive decisions. Our study outlines the detectability of anomalies in the first trimester in a primary care setting and raises awareness about potential missed anomalies. Given that many undetected anomalies led to TOP, our findings also highlight the importance of thorough parental counseling, informed consent and ethical reflection in early pregnancy care. The results of this study are important for setting realistic expectations among parents, healthcare staff and legal professionals, as the diagnostic performance of ultrasound examinations conducted in a comparable primary care setting is fundamentally different from that of scans executed by a dedicated team in a university hospital or private clinic. Potential improvements may be achieved through enhanced sonographer training, more stringent monitoring of the FTAS protocol, increased use of transvaginal scanning, protocol adjustments and incorporation of artificial intelligence.

### Strengths and limitations

This is the first study to analyze false‐negative cases following the introduction of the FTAS in a national primary care setting, and is one of the largest studies on this subject. It is likely that all undetected anomalies were included, since all centers for prenatal diagnosis in The Netherlands partook in the study. In addition, the protocol was applied uniformly and executed by certified sonographers. A limitation of this study is that we reported only on the first year following implementation of the FTAS; increasing experience with first‐trimester ultrasound may improve detection rates. Furthermore, we included only anomalies detected during the second‐trimester anomaly scan. Moreover, most hydrops and megacystis cases developed after the FTAS, and certain anomalies only became evident later in pregnancy. Finally, the mode of scanning used at the FTAS was unknown for the undetected cases evaluated herein; however, across the entire cohort of pregnancies that underwent a FTAS, the transvaginal route was used in only 14.7% of scans.

### Conclusions

Assessment of fetuses with a structural anomaly that remained undetected following FTAS within a nationally steered program showed that only a small proportion of FTMAs were missed. However, a considerable number of other major anomalies, including univentricular cardiac defects and spina bifida with a large defect, were overlooked in the first trimester. Image assessment showed that missing mandatory planes and acquisition of suboptimal planes by the sonographer were the main explanations for not detecting the abnormality. This overview of the undetected cases should inform the expectations of prospective parents and could provide a basis for improving performance of the FTAS.

## Supporting information


**Appendix S1** Structured form for evaluating image quality from first‐trimester anomaly scan, developed as part of the IMITAS study.

**Table S1** Characteristics of 26 fetuses with normal first‐trimester anomaly scan result that were ultimately diagnosed with structural anomaly that is considered often detectable in the first trimester and genetic aberration.
**Table S2** Classification of 1008 fetuses with structural anomaly that is considered undetectable in the first trimester that was not detected at first‐trimester anomaly scan, according to pregnancy outcome.
**Table S3** Characteristics of 52 fetuses with normal first‐trimester anomaly scan result that were ultimately diagnosed with structural anomaly that is considered undetectable in the first trimester and genetic aberration.

## Data Availability

The data that support the findings of this study are available from the corresponding author upon reasonable request.
