# Supplementary material for: Undetected cases after implementation of first‐trimester anomaly scan in low‐risk population: insights from the IMITAS study
Source: Ultrasound Obstet Gynecol. 2025 Nov 14;67(1):27–33. doi: 10.1002/uog.70131 (PMC12757820; doi:10.1002/uog.70131)
Supplement: Supplementary file 1 — Appendix S1 Structured form for evaluating image quality from first‐trimester anomaly scan, developed as part of the IMITAS study. [file UOG-67-27-s002.docx]

| **IMITAS image assessment FTAS** |
| --- |

| **IMITAS-ID** |  |
| --- | --- |
| **Name scorer** |  |
| **Date of assessment** |  |


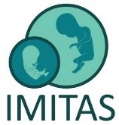


| **General information** |
| --- |
| \|  \|  \| \| --- \| --- \|  \|  \|  \| \| --- \| --- \|   **GA during FTAS:**  days  weeks |
| \|  \|  \|  \| \| --- \| --- \| --- \|   **Number of images:** |
| \|  \|  \| \| --- \| --- \|  \|  \|  \| \| --- \| --- \|   **Scan duration:**  minutes  hour |
| **Image resolution**  Good  Poor |

**Assessment of the FTAS images**

Tick the box for the number of points per image.

*FYI: per image, "good" may correspond to either 1 or 2 points.*

**Fill in 1 or 0 or a**  **Fill in 2 or 0 or a**

1 = good 2 = good

0 = insufficient 0 = insufficient

a = absent plane a = absent plane

| **Assessment of fetal anatomy** | | | |
| --- | --- | --- | --- |
| **Central nervous system** | | | |
| **Head & brain** | | Points | Key points |
| Assessment intactness and shape in transverse section  Assessment of “midline” presence and choroid plexus | Magnification | 1 –  0 –  a | - Covers 2/3 of the image |
|  | Section | 2 –  0 –  a | - Transverse section caput - Oval cranial shape - The midline as horizontal as possible in the image - Choroid plexuses filling lateral ventricles, forming a butterfly shape |
| **Spine** | | | |
| Assessment spine (sagittal section) en assessment continuity of the skin | Magnification | 1 –  0 –  a | - Covers 2/3 of the image |
|  | Section | 2 –  0 –  a | - Sacral to cervical region visible in the image - Continuity of the skin, separate from uterine wall - Midsagittal section |
| **Neck** | | | |
| Assessment nuchal translucency (NT-measurement) | Magnification | 1 –  0 –  a | - Fetal head, including the neck, displayed full-screen |
|  | Section | 2 –  0 –  a | - Midsagittal section of fetal head and neck in neutral position - Cranial contour visible as a clearly sharp white line - Occipital bone and fluid accumulation in the neck visible - Orbit not visible |
|  | Caliper placement | 2 –  0 –  a | - NT measurement at maximum thickness - Gain   *If the NT cannot be measured, the image where the NT is assessed should be recorded* |
| **Total score central nervous system**  Maximum 11 |  | \|  \| \| --- \| |  |
| **Profile** | | | |
| Assessment profile | Magnification | 1 –  0 –  a | - Covers 2/3 of the image |
|  | Section | 2 –  0 –  a | - Midsagittal section - Forehead, nasal bone, chin, and entire head visible |
| **Total score profile**  Maximum 3 |  | \|  \| \| --- \| |  |
| **Thorax en heart** | | | |
| **Thorax** | | | |
| Assessment of thoracic shape and lung appearance | Magnification | 1 –  0 –  a | - The thorax should occupy half of the image |
|  | Section | 2 –  0 –  a | - Transverse section at the level of the four-chamber view - Symmetrical, ribs straight - One rib visible |
| **Heart** | | | |
| Assessment of position and four-chamber view | Section | 2 –  0 –  a | - Transverse section of the thorax with four-chamber view - Symmetrical chambers |
| Assessment of separate filling of the ventricles with color Doppler (or power Doppler if color Doppler provides insufficient signal) | Magnification | 1 –  0 –  a | - |
|  | Section | 2 –  0 –  a | - |
|  | Color Doppler | 2 –  0 –  a | - Gain - Color box - PRF/velocity - Correct use of color Doppler of power Doppler |
| **Total score thorax en heart**  Maximum 10 |  | \|  \| \| --- \| |  |
| **Abdomen** | | | |
| Assessment abdominal wall and umbilical cord insection | Magnification | 1 –  0 –  a | - Covers 2/3 of the image |
|  | Section | 2 –  0 –  a | - Transverse section - Insertion visible |
| Assessment fluid-filled stomach (in AC-section) | Magnification | 1 –  0 –  a | - Covers 2/3 of the image |
|  | Section | 2 –  0 –  a | - Transverse section - Stomach visible |
| Assessment fluid-filled bladder | Magnification | 1 –  0 –  a | - Rump covers 2/3 of the image |
|  | Section | 2 –  0 –  a | - Longitudinal section - Bladder visible - If bladder appears to be enlarged: measure longitudinal diameter |
| **Total score abdomen**  Maximum 9 |  | \|  \| \| --- \| |  |
| **Extremities** | | | |
| Assessment right upper limb | Magnification | 1 –  0 –  a | - |
|  | Section | 1 –  0 –  a | - Visible connection between forearm and hand - Two bones forearm visible - Annotation right |
| Assessment left upper limb | Magnification | 1 –  0 –  a | - |
|  | Section | 1 –  0 –  a | - Visible connection between forearm and hand - Two bones forearm visible - Annotation left |
| Assessment right lower limb | Magnification | 1 –  0 –  a | - |
|  | Section | 1 –  0 –  a | - Visible connection between leg and foot - Two bones lower leg visible - Annotation right |
| Assessment left lower limb | Magnification | 1 –  0 –  a | - |
|  | Section | 1 –  0 –  a | - Visible connection between leg and foot - Two bones lower leg visible - Annotation left |
| **Total score extremities**  Maximum 8 |  | \|  \| \| --- \| |  |
| **Total fetal anatomy**  Maximum 41 |  | \|  \| \| --- \| |  |
| **Totaal number of ‘a’ fetal anatomy** |  | \|  \| \| --- \| |  |

| **Assessment of fetal biometrics** | | | |
| --- | --- | --- | --- |
| **Crown-rump length (CRL)** | | Points | Key points |
| Maximum 5 points | Magnification | 1 –  0 –  a | - Covers 2/3 of the image |
|  | Section | 2 –  0 –  a | - Fetus midsagittal - Fetus in neutral position, neither flexed or hyperextended |
|  | Caliper placement | 2 –  0 –  a | - Caliper 1 on skin of the crown and caliper 2 on skin of the tailbone - Focus set at the level of the fetus - Only measure until 84mm |
| **Head circumference (HC)** | | | |
| Maximum 2 points | Ellipse placement | 2 –  0 –  a | - Covers 2/3 of the image - Measurement without skin; calipers on the bone - Midline as horizontal as possible in the image |
| **Abdominal circumference (AC)** | | | |
| Maximum 2 points | Caliper placement | 2 –  0 –  a | - Covers 2/3 of the image - Include skin, spine and rib visible, umbilical vein at 1/3 of abdominal wall, kidneys not visible |
| **Femur length (FL)** | | | |
| Maximum 5 points | Magnification | 1 –  0 –  a | - Do not enlarge more than 2/3: sharp boundary |
|  | Section | 2 –  0 –  a | - Skin line of the upper leg parallel to the femur |
|  | Caliper placement | 2 –  0 –  a | - Distinctly visible, only bony diaphysis - If both femora are visible: measure the upper femur |
| **Total fetal biometrics**  Maximum 14 |  | \|  \| \| --- \| |  |
| **Total number of ‘a’ fetal biometrics** |  | \|  \| \| --- \| |  |

| **Conclusion**  To be completed by scorer | |
| --- | --- |
| **Conclusion** | Fetal anomaly is not suspected  Suspected fetal anomaly |
| *If an anomaly is suspected*  **Clarify type of anomaly** |  |
| **Comments** |  |
