# Supplementary material for: Undetected cases after implementation of first‐trimester anomaly scan in low‐risk population: insights from the IMITAS study
Source: Ultrasound Obstet Gynecol. 2025 Nov 14;67(1):27–33. doi: 10.1002/uog.70131 (PMC12757820; doi:10.1002/uog.70131)
Supplement: Supplementary file 2 — Table S1 Characteristics of 26 fetuses with normal first‐trimester anomaly scan result that were ultimately diagnosed with structural anomaly that is considered often detectable in the first trimester and genetic aberration. Table S2 Classification of 1008 fetuses with structural anomaly that is considered undetectable in the first trimester that was not detected at first‐trimester anomaly scan, according to pregnancy outcome. Table S3 Characteristics of 52 fetuses with normal first‐trimester anomaly scan result that were ultimately diagnosed with structural anomaly that is considered undetectable in the first trimester and genetic aberration. [file UOG-67-27-s001.docx]

**Supplementary Tables**

**Table S1** Characteristics of 26 fetuses with normal first-trimester anomaly scan that were ultimately diagnosed with ‘often detectable’ structural anomaly and genetic aberration

|  | **Total**  **N (%)** | **Findings at the SAS** | **Structural anomaly at diagnostic scan** |
| --- | --- | --- | --- |
| Trisomy 18 | 2 | MCA (n=1), heart (n=1) | MCA (n=1), heart (n=1) |
| Trisomy 21 | 8 | heart (n=5), MCA (n=1), brain (n=1)  abnormal fetal growth (n=1) | heart (n=4), MCA (n=2), brain (n=1), abnormal fetal growth (n=1) |
| Triploid | 1 | Abnormal fetal growth (n=1) | MCA (n=1) |
| Single gene disorders | 15 | Abnormal fetal growth (n=5), MCA (n=2), brain (n=2), heart (n=2), skeletal (n=2), extremities (n=1), face (n=1) | MCA (n=6), abnormal fetal growth (n=3), skeletal (n=2), brain (n=1), heart (n=1), face (n=1), thoracic (n=1) |
|  |  |  |  |
| **Total** | **26** |  |  |
| Abbreviations: MCA, multiple congenital anomalies; SAS, second-trimester anomaly scan. | | | |

**Table S2** Classification of 1008 fetuses with ‘undetectable’ structural anomaly that was not detected at first-trimester anomaly scan, according to pregnancy outcome

|  | **Total** | **Live birth** | **TOP** | **IUFD** | **Other/**  **unknown** |
| --- | --- | --- | --- | --- | --- |
| ***Central nervous system*** | **68** | **40** | **24** | **2** | **2** |
| Fossa posterior anomaly* | 11 | 7 | 4 | 0 | 0 |
| Intracranial cyst | 22 | 17 | 2 | 1 | 2 |
| Multiple intracranial anomalies | 3 | 1 | 2 | 0 | 0 |
| Corpus callosum agenesis | 24 | 9 | 15 | 0 | 0 |
| Other^†^ | 8 | 6 | 1 | 1 | 0 |
|  |  |  |  |  |  |
| ***Face*** | **71** | **68** | **3** | **0** | **0** |
| Cleft lip and/or palate | 71 | 68 | 3 | 0 | 0 |
|  |  |  |  |  |  |
| ***Neck***^‡^ | **5** | **5** | **0** | **0** | **0** |
|  |  |  |  |  |  |
| ***Thorax/lungs***^§^ | **28** | **26** | **2** | **0** | 0 |
| Congenital cystic adenomatoid malformation (CPAM/CCAM) | 27 | 26 | 1 | 0 | 0 |
| Other^§^ | 1 | 0 | 1 | 0 | 0 |
|  |  |  |  |  |  |
| ***Heart*** | **256** | **218** | **35** | **2** | **1** |
| Outflow tract anomalies^¶^ | 168 | 136 | 31 | 1 | 0 |
| Septal defect^**^ | 58 | 55 | 2 | 1 | 0 |
| Minor CHD^††^ | 30 | 27 | 2 | 0 | 1 |
|  |  |  |  |  |  |
| ***Abdomen*** | **124** | **123** | **0** | **0** | **1** |
| Intra-abdominal cyst | 28 | 28 | 0 | 0 | 0 |
| Esophageal atresia | 7 | 7 | 0 | 0 | 0 |
| Abnormal anatomy umbilical vein^‡‡^ | 56 | 56 | 0 | 0 | 0 |
| Echogenic focus^§§^ | 12 | 12 | 0 | 0 | 0 |
| Intestinal anomaly^¶¶^ | 15 | 15 | 0 | 0 | 1 |
| Gallbladder anomaly | 6 | 6 | 0 | 0 | 0 |
|  |  |  |  |  |  |
| ***Urogenital system*** | **258** | **249** | **6** | **1** | **2** |
| Urethral anomaly^***^ | 3 | 3 | 0 | 0 | 0 |
| Hydronephrosis | 53 | 53 | 0 | 0 | 0 |
| Multicystic or polycystic kidney(s) dysplasia | 40 | 37 | 3 | 0 | 0 |
| Renal agenesis | 30 | 26 | 3 | 1 | 0 |
| Ureteral duplication | 68 | 67 | 0 | 0 | 1 |
| Genital anomaly^†††^ | 14 | 14 | 0 | 0 | 0 |
| Pelvic / horseshoe kidney / unilocular cyst | 50 | 49 | 0 | 0 | 1 |
|  |  |  |  |  |  |
| ***Skeletal*** | **21** | **20** | **1** | **0** | **0** |
| Craniosynostosis | 1 | 1 | 0 | 0 | 0 |
| Pectus excavatum | 3 | 3 | 0 | 0 | 0 |
| Hemivertebrae | 5 | 5 | 0 | 0 | 0 |
| Sacrococcygeal teratoma | 5 | 5 | 0 | 0 | 0 |
| Other^‡‡‡^ | 7 | 6 | 1 | 0 | 0 |
|  |  |  |  |  |  |
| ***Extremities*** | **129** | **121** | **5** | **1** | **2** |
| Pes equinovarus | 108 | 102 | 3 | 1 | 2 |
| Limb deformities^§§§^ | 3 | 3 | 0 | 0 | 0 |
| Abnormal limb position (excl. pes equinovarus) ^¶¶¶^ | 6 | 4 | 2 | 0 | 0 |
| Deformities of fingers/toes^***^ | 10 | 10 | 0 | 0 | 0 |
| Other^††††^ | 2 | 2 | 0 | 0 | 0 |
|  |  |  |  |  |  |
| ***MCA*** | **48** | **26** | **19** | **2** | **1** |
|  |  |  |  |  |  |
| **Total (%)** | **1,008 (100.0)** | **896 (88.9)** | **95 (9.4)** | **8 (0.8)** | **9 (0.9)** |
| Abbreviations: MCA, multiple congenital anomalies.  *Fossa posterior anomaly (incl. cerebellar anomalies): hemorrhage, Dandy-Walker syndrome, Blake’s pouch, cerebellar hypoplasia, cyst in fossa posterior.  †Nervous system other: cavum velum interpositum, vein of Galen malformation, dural sinus malformation, septed or absent cavum pellucidum (CSP), partial holoprosencephaly.  ^‡^Neck: fetal struma, lateral neck cyst, lymphangioma, absent thymus.  ^§^Thorax/lungs other: sternal echogenic spots  ^¶^Outflow tract anomalies: Tetralogy of Fallot, transposition of the great arteries (TGA), truncus arteriosus, pulmonary atresia with VSD, aortic arch anomalies, pulmonary and aortic stenosis.  ^**^Septal defects: ventricular septal defect(s) (VSD).  ^††^Minor congenital heart disease (CHD): e.g. venous return anomalies, left/right disproportion, aneurysm foramen ovale, isolated cardiac malposition, pericardial cyst.  ^‡‡^Abnormal anatomy umbilical vain: persistent right umbilical vein (PRUV), varix.  ^§§^Echogenic abdominal focus: e.g. liver calcifications (excl. echogenic bowel).  ^¶¶^Intestinal anomaly: dilated bowel, duodenum atresia, anorectal malformation.  ^***^Urethral anomaly: megalourethra, diverticulum.  ^†††^Genital anomaly: hypospadias, ambiguous, differenced in sex development (DSD).  ^‡‡‡^Skeletal other: lemon sign without spina bifida, human tail, supernumerary sacral vertebrae, scoliosis.  ^§§§^Limb deformities: e.g. hypoplasia femur, radius, ula.  ^¶¶¶^Abnormal limb position: e.g. abnormal position of hands and/or feet, arthrogryposis, rocker-bottom feet.  ^****^Deformities of fingers/toes: syndactyly, polydactyly.  ^††††^Extremity other: peripheral edema | | | | | |

**Table S3** Characteristics of 52 fetuses with normal first-trimester anomaly scan that were ultimately diagnosed with ‘undetectable’ structural anomaly and genetic aberration

|  | **Total**  **N (%)** | **Findings at the SAS** | **Structural anomaly at diagnostic scan** |
| --- | --- | --- | --- |
| Trisomy 18 | 6 | MCA (n=5), heart (n=1) | MCA (n=5), heart (n=1) |
| Trisomy 21 | 2 | Heart (n=2) | Heart (n=2) |
| Monosomy X | 1 | Heart (n=1) | Heart (n=1) |
| 22q11 deletion syndrome | 9 | Heart (n=9) | Heart (n=9) |
| Single gene disorders | 34 | Heart (n=11), brain (n=7), MCA (n=5), extremities (n=4), skeletal (n=2), abdomen (n=1), face (n=1), neck (n=1), thoracic (n=1), urogenital (n=1) | Heart (n=10), brain (n=7), MCA (n=6), extremities (n=3), neck (n=2), skeletal (n=2), abdomen (n=1), face (n=1), thoracic (n=1), urogenital (n=1) |
|  |  |  |  |
| **Total** | **52** |  |  |
| Abbreviations: MCA, multiple congenital anomalies; SAS, second-trimester anomaly scan. | | | |
